# Targeting Endotypic Traits with Medications for the Pharmacological Treatment of Obstructive Sleep Apnea. A Review of the Current Literature

**DOI:** 10.3390/jcm8111846

**Published:** 2019-11-02

**Authors:** Luigi Taranto-Montemurro, Ludovico Messineo, Andrew Wellman

**Affiliations:** 1 Brigham and Women’s Hospital and Harvard Medical School, Boston, MA 02115, USA; awellman@bwh.harvard.edu; 2 Adelaide Institute for Sleep Health, Flinders University, Adelaide 5001, Australia; ludovico.messineo@yahoo.it

**Keywords:** OSA phenotypes, OSA endotypes, OSA pharmacotherapy, precision medicine

## Abstract

Obstructive sleep apnea (OSA) is a highly prevalent condition with few therapeutic options. To date there is no approved pharmacotherapy for this disorder, but several attempts have been made in the past and are currently ongoing to find one. The recent identification of multiple endotypes underlying this disorder has oriented the pharmacological research towards tailored therapies targeting specific pathophysiological traits that contribute differently to cause OSA in each patient. In this review we retrospectively analyze the literature on OSA pharmacotherapy dividing the medications tested on the basis of the four main endotypes: anatomy, upper airway muscle activity, arousal threshold and ventilatory instability (loop gain). We show how recently introduced drugs for weight loss that modify upper airway anatomy may play an important role in the management of OSA in the near future, and promising results have been obtained with drugs that increase upper airway muscle activity during sleep and reduce loop gain. The lack of a medication that can effectively increase the arousal threshold makes this strategy less encouraging, although recent studies have shown that the use of certain sedatives do not worsen OSA severity and could actually improve patients’ sleep quality.

## 1. Introduction

Obstructive sleep apnea (OSA) is a common and harmful medical condition that remains undertreated due to poor compliance with the leading therapy, continuous positive airway pressure (CPAP) [1,2]. It is estimated that approximately 15% of males and 5% of women in North America are affected by this disorder [3], and recently, a global estimate suggested that more than 930 million people suffer from at least mild OSA [4]. Currently, there is no approved pharmacotherapy for OSA and the research in this field has to date been disappointing. Nevertheless, recent developments related to an improved understanding of OSA pathophysiology [5] and to the identification of specific pharmacological targets [6] has brought new hope to the field for treating this disorder with medications.

### Personalized Medicine Approach

**Figure 1:**
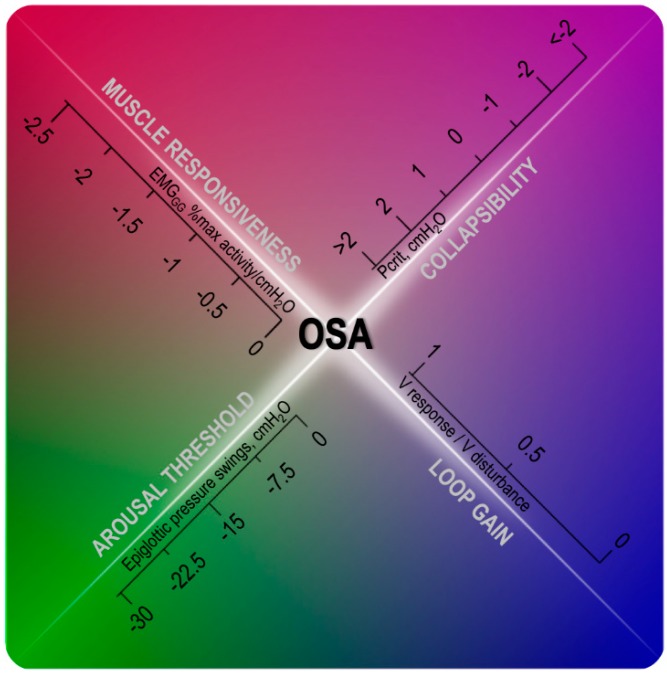
Diagram showing the interaction of four endotypic traits on obstructive sleep apnea (OSA) pathogenesis: in the presence of mild-to-moderate collapsibility, indicated by a critical collapsing pressure (Pcrit) between −2 and 2 cmH_2_O, other non-anatomical traits play a role in OSA pathophysiology. The inability to recruit the upper airway dilator muscle in response to negative pharyngeal pressure swings during an obstructive event (% activity/cmH_2_O close to 0), a low arousal threshold (epiglottic pressure swings before the arousal above −15 cmH_2_O) and a high loop gain (close to or above 1) will contribute in different degrees to OSA development. Note that the boundaries between the four traits are intentionally blurred to show that OSA presence and severity is often determined by the interaction of these pathogenic traits. EMG_GG_: genioglossus electromyography, V response/V disturbance: ratio between the ventilatory response to a preceding ventilatory disturbance, dimensionless.

In the last 15 years, researchers have shown that a number of key pathophysiological traits, or endotypes, contribute to the pathogenesis of OSA [5,7,8,9,10,11] (Figure 1). These include (1) a small, collapsible upper airway (2) reduced dilator muscle responsiveness, (3) reduced arousal threshold, and (4) an oversensitive ventilatory control system (high loop gain). CPAP is efficacious in most pathophysiological conditions, i.e., regardless of the underlying endotypic traits, because it can completely abolish the obstructive events by splinting the airway open. To increase the chances of success, the pharmacological approach needs instead to target specific mechanisms causing OSA, thus, a better understanding of the patient-specific etiology of this disorder is essential. It has been estimated that anatomical defects are the main cause of OSA in about 46% of patients, insufficient upper airway muscles recruitment plays an important role in approximately 36%, and a high loop gain in 36% [5]. A low arousal threshold was found in 88%, 73% and 23% of patients with mild, moderate and severe OSA, respectively, suggesting that this endotype is more important in patients with a mild rather than a severe disorder [12]. These estimates need to be interpreted cautiously because are derived from a limited cohort of OSA patients from a single research laboratory.

While this endotype-based approach has so far been limited to the research setting, recent progresses have allowed measurements of the traits from the clinical polysomnography and will possibly be included in future software packages [13,14,15]. Measuring the change in endotypic traits with an intervention will let researchers and physicians to go beyond the usual metrics of OSA severity, the apnea-hypopnea index (AHI) or the respiratory disturbance index (RDI). To date, the AHI is the main parameter used in research and clinical studies, and it informs the physician about the necessity for treatment. However, despite its widespread use, the AHI correlates poorly with subjective symptoms such as fatigue or sleepiness [16], as well as other health outcomes [17].

The efficacy of medications on OSA severity has been evaluated using AHI as the main outcome. Oxygen desaturation, subjective symptoms and effects on endotypic traits were also used, although less frequently. In the near future, thanks to developing technologies, we also hope that the efficacy of new pharmacotherapies and alternative treatments will be tested on other metrics, such as the hypoxic burden that better characterizes the ventilatory burden of sleep apnea [17] and its consequences.

Here we review evidence for the efficacy of medications tested for OSA. We divided the medications into different classes based on the mechanism of action and according to the four aforementioned endotypes (i.e., anatomy, upper airway muscle activation, arousal threshold and loop gain). Most studies enrolled unselected volunteers with OSA, and thus the efficacy of these same medications may be higher if administered to selected individuals according to their endotype. A summary of the tested drugs can be found for each group in a figure where the efficacy of the medications is compared using a weighted-average of the data available from different studies. We included in this review all the available randomized-controlled trials or observational studies performed in adults testing the effect of medications on the AHI and reported in English language. Studies investigating central sleep apnea or OSA at high-altitude were not analyzed in this review. Investigations testing drugs that had no measured or theoretical relationship with the four endotypic traits (i.e., β-blockers or proton pump inhibitors) were also excluded. It must be noticed that some drugs tested do not fit uniquely in a single endotype category: for example, topiramate is used for weight loss therapy and is also a carbonic anhydrase inhibitor that may influence ventilatory control stability [18], and oxybutynin, that enhances upper airway muscles activity, may also have sedative effects [19].

## 2. Upper Airway Anatomy

**Figure 2:**
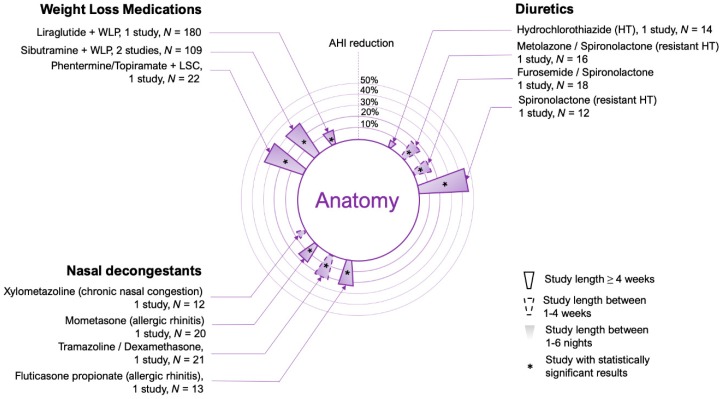
Circular bar plot describing the studies performed using medications that modify anatomic traits in OSA patients. Only observational studies and clinical trials in which the apnea-hypopnea index (AHI) or the respiratory disturbance index (RDI) was reported were considered. No case reports or case series were included. AHI reduction refers to percent change from placebo values (or baseline when placebo was not available). Data represents a weighted average of AHI reductions if more than one study was testing the same medication(s). Study length (see legend) refers to the longest study performed using a particular medication. Every asterisk represents one single significant study. See text for specific differences and details of the studies considered. HT: hypertension.

All patients with OSA share some anatomic predisposition to upper airway collapse. Possible causes of this anatomic susceptibility include fat deposition in the tissue surrounding the airway due to obesity [20], a reduced bony skeletal structure [21], increased tonsil or adenoid size [22] or upper airway edema due to fluid retention [23]. Chronic nasal congestion (rhinitis) may also increase the likelihood of pharyngeal collapse due to the generation of more negative pressure in the downstream segment of the upper airway [24]. Targeting anatomy with medical therapy can be challenging. For example, micrognatia or tonsillar hypertrophy necessarily needs surgical interventions. Nevertheless, a number of medications could help to improve anatomy in selected patients: weight loss drugs, diuretics, and nasal decongestants (see Figure 2 for a summary of the drugs tested in this category).

### 2.1. Weight Loss Medications

Weight loss has recently been introduced into the guidelines for the treatment of OSA, since obesity is the most important risk factor for this disorder [25]. The prevalence of OSA in the obese population is about 50% [26], and body mass index (BMI) is linearly related with OSA severity. However, a recent metanalysis on the effect of surgical weight loss on OSA [27] showed that there is no relationship between the amount of weight loss and AHI reduction in these patients likely because other factors are at play in determining the AHI even when the anatomical defect has been reduced. According to this metanalysis, despite frequent complications, bariatric surgery showed an average 28% weight reduction, with a consequent reduction in AHI by 68% in severe OSA patients [27]. Another recent metanalysis showed that weight loss programs including lifestyle changes (without medications or surgery) can lead to ~13% of weight loss in about 12 months with an average reduction in AHI by 48% in moderate OSA patients [28,29]. One trial from Tuomilehto et al. [30], suggested the possibility that the improvements in AHI with weight loss may not be persistent over time and more data need to be collected in longitudinal studies.

To help the fight to obesity, in the last 10 years, four weight-loss drugs have been approved by the US Food and Drug Administration and some of them have already been tested in patients with OSA. In an uncontrolled cohort study, Yee et al. [31] tested the effect of sibutramine, a serotonin and norepinephrine reuptake inhibitor, in combination with a weight loss program, in 87 middle-aged obese patients with OSA. After six months, the RDI was reduced by 35% compared to baseline, along with a 10% weight loss and improvement in subjective sleepiness. A subsequent smaller, open label trial by Ferland et al. [32] compared treatment with sibutramine and dietary restriction in 22 OSA patients vs CPAP in 18 patients for one year. Weight loss in the sibutramine group was only 5% from baseline and was insufficient to cause a significant reduction in AHI from baseline.

Liraglutide, a glucagon-like peptide-1 analog approved for type-2 diabetes and weight loss, was tested by Blackman et al. [33] in a 8-months double-blind trial vs. placebo in a group of 180 vs. 179 moderate-to-severe OSA patients, respectively. The liraglutide group lost 4.2% more weight and had a 12% more reduction in AHI compared to placebo.

Finally, in a double-blind, placebo-controlled trial, Winslow et al. [18] tested the combination of phentermine, a sympathomimetic agent, and topiramate, a GABAergic sedative and carbonic anhydrase inhibitor, on OSA severity. Both arms also had lifestyle modification counseling. Fifty-five patients (22 in the medications arm and 23 on placebo) took part to the 28-week trial. In the drugs group, the patients lost 5.9% more weight (−10.2% from baseline) and their AHI was 36% lower compared to placebo (−71% from baseline of 44.2 events/h) suggesting that this combination could be very helpful in treating obese OSA patients.

### 2.2. Upper Airway Edema

It has been hypothesized that fluid volume regulation and distribution may be involved in the pathogenesis of OSA [23,34]. Several studies have shown a high prevalence of OSA in fluid-retaining states including resistant hypertension [35], heart failure [36] and end-stage renal disease [37]. The fluid accumulation in the body may lead to increased parapharyngeal edema and upper airway collapsibility, due to gravity-driven fluid redistribution towards the neck from the lower extremities when laying down [38].

Medications targeting fluid retention, including diuretics and mineralocorticoid antagonists, have shown potential for reduction of OSA severity, at least in subgroups of patients. In particular, the mineralocorticoid inhibitor spironolactone has emerged as a potential agent for OSA treatment when administered alone or in combination with other diuretics. In a group of 12 moderate-to-severe OSA patients with resistant hypertension (i.e., ≥3 antihypertensives), Gaddam et al. [39] added spironolactone and measured the effect on AHI after eight weeks of treatment. In this open label study there was a reduction in AHI by 45% from baseline together with improvement in oxygen desaturations and reduced blood pressure. Furthermore, Kasai et al. [40] tested the effect of spironolactone with metolazone on OSA severity in a two-week prospective observational study in 16 patients with resistant hypertension. In this study, the adjunctive treatment caused a 16% reduction in AHI from baseline (57.7 to 48.5 events/h). Finally, in another recent three-parallel arm randomized trial by Fiori et al. [41], the combination of furosemide and spironolactone was administered to 18 OSA patients without resistant hypertension and compared to sodium-restricted diet (*N* = 18) or placebo (*N* = 18). The combination of diuretics caused a 16% reduction in AHI, while the sodium-restricted diet reduced it by 24% compared to placebo. Therefore, in the right populations diuretics such as spironolactone appear have a modest effect on AHI.

### 2.3. Nasal Decongestants

High nasal resistance can contribute to pharyngeal collapse by increasing the negative suction pressure downstream in the velo- and oropharynx. Indeed, a recent study demonstrated an OSA prevalence as high as 65% in patients with chronic rhinosinusitis [24]. Therefore, a drug that reduces nasal congestion could potentially improve OSA in some patients.

Several nasal decongestants have been trialed for OSA severity. The effect on AHI of mometasone alone and in combination with the antihistaminic desloratadine was measured in patients with allergic rhinitis by Acar et al. [42] in a four-arm (mometasone, desloratadine, mometasone + desloratadine and placebo) trial with 80 patients. While desloratadine alone or in combination with mometasone did not show significant improvement of OSA severity, mometasone alone did, with a reduction in AHI by 17% compared to placebo.

Another nasal steroid, fluticasone, was tested in 13 patients with rhinitis and OSA for four weeks in a randomized, double blind, placebo controlled, crossover study by Kiely et al. [43]. Treated patients showed a 21% reduction in AHI compared to placebo. By contrast, a recent parallel-arm trial performed by Smith et al. testing the combination of fluticasone and montelukast in patients with mild OSA alone (without rhinitis) did not find any difference in AHI between groups, although total sleep time and rapid eye movement (REM) sleep were increased in the treatment arm, suggesting a possible improvement in sleep quality related to reduced nasal resistance [44].

The combination of the nasal steroid dexamethasone with the decongestant tramazoline was tested by Koutsourelakis et al. [45] in a group of 21 OSA patients with normal nasal resistance in a randomized, double-blind, placebo-controlled, crossover trial of one week duration. The treatment reduced the AHI by 20% compared to baseline (16% compared to placebo). Interestingly, the patients reduced mouth breathing on treatment, and the increase in nasal breathing was proportional to the reduction in AHI. This is consistent with previous findings suggesting that mouth opening (and consequently mouth breathing) is associated with increased upper airway collapsibility and total respiratory resistance [46]. The nasal decongestant xylometazoline was also tested on OSA severity by Clarenbach et al. [47] in 12 patients with chronic nasal congestion in a similar crossover trial lasting one week. Although the drug reduced nasal resistance overnight, the AHI was only reduced by 18% in the first part of the polysomnography (i.e., when the medication effect was likely more pronounced), but there was no effect on OSA severity when the entire night was taken into account.

Overall, these data show that patients with nasal congestion treated with topical corticosteroids may improve their sleep apnea, especially if they suffer from chronic rhinitis. More data are needed to confirm these findings, and studies using more potent anti-inflammatory agents (i.e., selective monoclonal antibodies) are ongoing [48].

## 3. Upper Airway Dilator Muscle Activation

In humans, there is no fixed bone or cartilage supporting the pharynx. Rather, it is held open by activation of the surrounding musculature. Relaxation of these muscles during sleep and lack of sufficient reactivation are key primary pathophysiological events leading to OSA [49]. Patients with OSA show higher activation of upper airway muscles during wakefulness compared to healthy controls; presumably in order to maintain a patent upper airway while awake. At sleep onset, however, there is a physiologic reduction in upper airway dilator muscle activity that occurs in all individuals [50,51]. This, together with impaired anatomy and/or unstable control of breathing, often leads to OSA during lighter stages of sleep. Epiglottic pressure swings and CO_2_ increase with deeper stages of sleep and during obstructive events [52], restoring pharyngeal muscle activity and, consequently, upper airway patency by reflexive recruitment. However, this reflex is widely variable between individuals, with some showing effective recruitment and the ability to partially reopen the upper airway, and others showing minimal or no upper airway muscle compensation during sleep [53,54].

**Figure 3:**
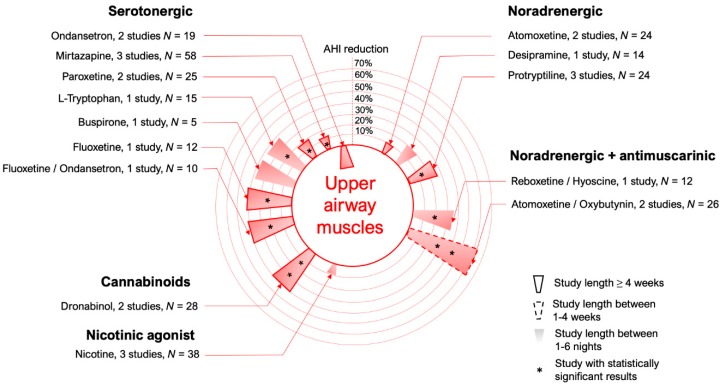
Circular bar plot describing the studies performed using medications that modify upper airway muscle activity.

Since the activity of upper airway dilator muscles is regulated by specific neurotransmitters whose concentration varies between wake and sleep states, it should be theoretically possible to manipulate the airway muscle tone by identifying these monoamines responsible for the muscle activation and administering them to OSA patients during sleep, thus preventing upper airway muscle relaxation. In the last decade, many advancements in basic science helped to refine the choice of receptor targets to stimulate the upper airway muscles, and attempts to translate these findings into OSA patients are still ongoing [6] (see Figure 3 for a summary of the drugs tested in this category to date).

### 3.1. Serotonergic Mechanisms

Withdrawal of serotonin at the hypoglossal nucleus was long thought to be the primary mechanism underlying decreased pharyngeal muscle activity during sleep [55,56]. The complexity of the serotonergic system is represented by the presence of seven different receptor families that are distributed in both the peripheral and central nervous systems and can be either excitatory or inhibitory [56].

Serotonin has shown clear excitatory effects on respiration when administered centrally in mammals. This excitation is mediated by receptors 5-HT2a/c on upper airway motorneurons and 5-HT1a receptors on respiratory neurons [57,58]. Serotonergic drive is diminished centrally from wakefulness to non-REM (NREM) sleep and it is minimal during REM sleep, leading to a relative reduction in ventilatory drive. This reduced 5-HT activity may contribute to upper airway collapse in patients with OSA. In contrast to predominantly excitatory central effects of serotonin on respiratory control, the effects of serotonin peripherally are principally inhibitory, and involve again 5-HT2a/c and 5-HT3 receptor subtypes. In summary, while 5-HT2a/c have different effects on respiration centrally and peripherally, 5-HT3 antagonists and 5-HT1a agonists consistently appear to improve respiration [56].

Ondansetron, an antagonist of 5-HT3, reduced by 54% the sleep disordered breathing in English bulldogs [59] but had no effect in 10 OSA patients in a single-night crossover trial at the dose of 16 mg. These data suggest that species differences or other causes (i.e., the low dose tested or the short time frame of the study) may be at play [60]. A pilot trial using buspirone, a 5-HT1a agonist in 5 OSA patients was published in the form of a letter by Mendelson et al. [61]. It showed an improvement in four out of five patients’ AHI vs placebo (the mean reduction for the whole group was 36%), an increased total sleep time, and a tendency for improvement in subjective sleep quality. Besides this small trial, no other data are available regarding buspirone in OSA to our knowledge.

Probably one of the most interesting serotonergic agents tested in OSA was mirtazapine, a 5-HT3 antagonist that showed initial positive results on 12 patients (50% reduction in AHI) after one-week administration [62]. These results were not confirmed in two subsequent trials of two and four weeks on 20 and 26 patients, respectively. In these studies mirtazapine did not show a significant reduction in OSA severity and was associated to a significant weight increase of 1.4 kg at four weeks [63].

Selective serotonin reuptake inhibitors (SSRIs) have the potential to reduce OSA severity by increasing the central concentration of serotonin that could ultimately bind to 5-HT receptors. Paroxetine and fluoxetine were both tested in two small trials. Paroxetine was first evaluated in a double blind, placebo-controlled crossover trial by Berry et al. [64] on eight patients in which the main outcome was genioglossus activity. Paroxetine significantly increased peak genioglossus muscle activity and muscle responsiveness (by 27%) compared to placebo during the first treatment night. However, the AHI remained unchanged. A subsequent study tested the same medication on 17 patients in a similar crossover design and showed a significant reduction in AHI by 18% from placebo over a period of six weeks. Specifically, the drug reduced the AHI during NREM sleep (35% reduction) but not during REM sleep, suggesting that two different mechanisms might be at play in these sleep stages. The most successful results with a SSRI were obtained in two trials testing fluoxetine. Hanzel et al. first compared the effects of four weeks of fluoxetine vs four weeks of protriptyline in 12 patients in an open-label, randomized crossover trial [65]. Both drugs significantly reduced the AHI by approximately 40% from baseline, but they did not improve oxygen desaturation, and there was large variability in the response among individuals. Fluoxetine was subsequently tested by Prasad et al. [66] in combination with ondansetron in 10 patients in a parallel group study. It showed, again, an AHI reduction of 40% compared to placebo after four weeks of treatment. Since ondansetron alone did not have an effect on AHI, we can speculate that most, if not all, of the result of the combination was due to fluoxetine, although a synergistic effect of the two drugs cannot be excluded.

L-tryptophan, an alpha-amino acid that serves as a precursor for serotonin synthesis, has been tested in an uncontrolled study in 15 sleep apnea patients (12 with OSA and three with central sleep apnea). The drug was administered before bedtime for one night and showed a 35% reduction in OSA severity [67]. Veasey et al. [68] confirmed the usefulness of tryptophan in an animal model (five English bulldogs), finding that, in association with the serotonergic antidepressant trazodone (discussed in the next section on arousal threshold) it decreased the AHI in both NREM and REM sleep and minimized sleep-related suppression of upper airway dilators. However, given the multiple side effects of tryptophan (i.e., eosinophilia-myalgia syndrome and pulmonary diseases [69]), L-tryptophan is no longer available on the US market.

### 3.2. Noradrenergic Mechanisms

The limited effectiveness of serotonergic drugs in OSA required a second look at the data obtained in animal models. It was recognized that some of the studies implicating serotonin were conducted in vagotomized animals, a common practice in pharyngeal motor control studies as vagotomy releases the genioglossus from inhibitory vagal afferents. Since vagal afferents inhibit serotonin at the hypoglossal nucleus, vagotomy could overestimate the central excitatory influence of endogenous serotonin [55]. Subsequent studies in animals with an intact vagus nerve [70,71,72] showed that withdrawal of endogenous serotonin has smaller effects on upper airway muscle activation than previous studies suggested. This could partly explain why serotonergic drugs administered to humans had minimal effects on OSA severity [64,65,73]. Moreover, evidence from Fenik and colleagues has shown that the loss of noradrenergic activity plays a key role in the sleep-related reduction of pharyngeal muscle activity, with serotoninergic mechanisms accounting for only about 10% [72] of the reduction. Indeed Chan and colleagues [74] showed that the noradrenergic antagonist terazosin administered at the hypoglossal motor nucleus in the brainstem of rats substantially reduced genioglossus activity during wakefulness and sleep, highlighting the importance of endogenous noradrenergic withdrawal.

Translational work performed recently in our laboratory showed that drugs with noradrenergic stimulant properties such as desipramine can increase genioglossus muscle activity [75] and reduce upper airway collapsibility during sleep in humans [54]. However, our team and others have also shown that, when taken alone, noradrenergic drugs such as norepinephrine reuptake inhibitors only mildly reduce OSA severity, and only in selected patients.

The tricyclic antidepressants protriptyline [76,77] and desipramine [54], as well as the selective norepinephrine reuptake inhibitor atomoxetine [78], have all been tested in patients with OSA, with modest success in reducing the severity of the disorder. Three randomized controlled trials [65,76,79] and several observational studies [77,80,81] assessed the effects of protriptyline on OSA severity. Brownell and co-workers [76] found no change in AHI during NREM sleep after four weeks of therapy with protriptyline 20 mg in a group of five obese men with severe OSA. However, the patients had improvements in oxygen saturation and daytime sleepiness, suggesting at least some positive impact of the drug on respiration during sleep. In another double-blind trial, Whyte and coworkers [79] found that administration of protriptyline 20 mg for 14 days in 10 moderate-to-severe OSA patients did not change NREM AHI as a group, but the inter-individual variability in response was substantial. Lastly, in an open label, four-week crossover trial on nine patients, Hanzel et al. [65] showed a statistically significant reduction in AHI by 42% from baseline. These results, taken together, suggest that protriptyline may be helpful in a subgroup of OSA patients that still needs to be identified.

Similar to protriptyline, another tricyclic drug, desipramine, was tested by our group in a placebo-controlled, double blind crossover trial lasting one night. However, variable results were obtained in terms of AHI reduction in the 14 patients studied [54]. Although the effect on OSA severity was not significant as a group, patients exhibited a less collapsible airway on desipramine compared to placebo. Moreover, a post-hoc analysis identified the subgroup of patients with minimal muscle compensation as the phenotype that responded best to the treatment. Additionally, in a trial performed on normal controls, desipramine increased genioglossus activity and reduced upper airway collapsibility during sleep [75].

The selective norepinephrine reuptake inhibitor atomoxetine was tested by Bart-Sangal et al. [78] in a prospective observational study of 15 patients with mild OSA. The drug did not improve AHI but did significantly improve daytime sleepiness. A second trial performed by our group confirmed that atomoxetine administered alone did not improve OSA severity in a sample of nine moderate-to-severe OSA patients [82].

According to Richard Horner’s group in Toronto, noradrenergic withdrawal is not the only mechanism involved in sleep-related loss of genioglossus activity. For example, noradrenergic stimulation with phenylephrine at the hypoglossal motor nucleus (HMN) failed to reverse REM sleep-related tongue muscle atonia [74], suggesting that an additional inhibitory mechanism might be at work. This inhibitory mechanism was identified in a follow up study to be predominantly muscarinic. Grace et al. delivered the muscarinic receptor antagonist scopolamine into the HMN in rats and demonstrated that there is progressive muscarinic inhibition of drive to the HMN from wakefulness to NREM and REM sleep. Muscarinic receptor antagonism had a particularly strong restorative effect on genioglossus activity in REM sleep. These findings have been recently applied to humans in a preliminary proof-of concept study by our laboratory. In 20 OSA patients, the combination of atomoxetine and the antimuscarinic oxybutynin was administered before bedtime for one night and compared to placebo [82]. Atomoxetine-plus-oxybutynin increased by ~three-fold the genioglossus muscle responsiveness to negative esophageal pressure swings and lowered the AHI by 63%. The combination reduced the AHI in both REM and NREM sleep, and there was an improvement in oxygen saturation parameters as well. These findings were replicated in six selected OSA patients in a subsequent open-label study performed in our laboratory after one week of therapy. A similar combination consisting of the adrenergic agent reboxetine and the anti-muscarinic drug hyoscine butylbromide was tested by Lim and coworkers on 12 healthy subjects in a double-blind, placebo-controlled, randomized, cross-over fashion [83]. The combination improved the activity of the tensor palatini muscle, a representative tonic upper airway muscle, and reduced pharyngeal resistance during sleep. However, it did not increase the phasic activity of the genioglossus muscle. Nevertheless, when these drugs were tested on a sample of 12 OSA subjects, the AHI decreased by approximately 35% from placebo, with a concurrent significant increase in the nadir oxygen saturation [84]. The results obtained in these trials are promising for the field of OSA pharmacotherapy and should be followed up with longer studies in a larger number of individuals.

### 3.3. K^+^ Channel Blockers

The modulation of K^+^ channels has been identified as the downstream mechanism through which wake-activating neurotransmitters such as norepinephrine, serotonin and acetylcholine modify the motorneuron membrane excitability in both NREM and REM sleep [85]. The opening of K^+^ channels produces a hyperpolarization of neurons due to increased K^+^ leak from the intracellular space, thereby reducing cell excitability [86].

Studies performed by Grace et al. in freely behaving rats have shown that blockade of potassium channels (i.e., promoting membrane depolarization and cell excitability) with several agents (barium, 4-aminopyridine (4-AP), and tetraethylammonium injected directly into the brainstem) leads to increased genioglossus activity during NREM and REM sleep [87]. However, potassium channels are ubiquitous in human cells, and the lack of a specific, “druggable” target on the HMN limits the possibilities for targeting this mechanism for treatment. Nevertheless, Suratt et al. [88] tested the effect of one-night intravenous administration of doxapram, a K^+^ channel inhibitor used as a respiratory stimulant, in a double-blind placebo-controlled trial in four patients. They found reductions in apnea and hypopnea length and improvement in oxygen desaturation on the drug night. In 10 healthy individuals, we evaluated the effect of 4-AP administered for a single night before bedtime on genioglossus muscle activity [89]. While the results were negative (there was no improvement in upper airway collapsibility), genioglossus activity was higher during REM sleep on 4-AP compared to placebo. Moreover, seven out of 10 patients had an improvement in genioglossus responsiveness to negative epiglottic pressure swings, even though the study was underpowered to show statistical significance on this outcome. Lastly, Bayer Pharmaceuticals is testing a K^+^ channel blocker to be administered topically in the upper airway in OSA patients in an ongoing phase-two clinical trial (BAY2253651) [90].

Ideally, targeting more specific K^+^ receptors that are entirely or almost exclusively expressed on brainstem motor neurons, such as the recently identified Kir 2.4, would allow a safer and possibly more effective approach for OSA patients [91].

### 3.4. Cannabinoids

There has also been recent interest in targeting cannabinoid receptors for treating OSA, which are functionally coupled to K^+^ channels [91,92]. Cannabinoid receptor (CBR) 2 is the third most strongly expressed G-protein coupled receptor in the HMN; it is expressed nearly two-fold more than adrenergic and muscarinic receptors [6]. Dronabinol is a partial agonist for CBR1 and 2, and it was recently tested in a proof-of-concept study followed by a subsequent phase-two clinical trial. In the initial experiment, 17 patients with moderate-to-severe OSA took increasingly higher doses of dronabinol (from 5 to 10 mg) for 21 days. Data collected on night 21 showed a significant reduction in AHI of 29% [93]. In the phase-2, parallel-arm, randomized controlled trial, 73 OSA patients with an AHI > 15 events/h received a placebo or dronabinol at low (*N* = 25) or high doses (*N* = 21). While this study showed a reduction in AHI of four events/h from baseline, it nevertheless constituted a significant decrease because of an increase in AHI of eight events/h in the placebo group. In a post-hoc analysis adjusted for baseline AHI, age, gender and race, the authors found that dronabinol at high doses reduced the AHI by 8.5 events/h (−33%). The patients also showed improvement in subjective daytime sleepiness, but oxygen desaturation was unchanged [94]. More data needs to be collected using dronabinol and other available cannabinoids to better understand their mechanisms of action and if they can improve OSA severity at least in a subgroup of patients.

### 3.5. Nicotine

It is known from animal studies that nicotine has stimulant effects on the genioglossus muscle and on ventilatory drive [95]. Based on these properties, it was hypothesized that nicotine could be useful for OSA treatment. Nicotine was tested in three small trials. Gothe et al. [96] showed that nicotine, administered via chewing gum (14 mg) to eight OSA patients before bedtime, reduced the time spent in apnea during the first hour of sleep by 20% and the number of apneas in the first 2 h of sleep. Davila et al. [97] subsequently administered nicotine patches (total dose 11 mg) to 20 patients with OSA in a randomized, controlled, crossover trial and found no significant difference in AHI compared to placebo. Finally, Zevin et al. [98] administered 2 and 4 mg of nicotine via a tooth patch over two different nights in a prospective observational study in 10 OSA patients. They found no differences in OSA severity with any of the tested doses. These studies indicate that the short half-life and the small effect size measured with nicotine do not encourage further its clinical investigation.

## 4. Arousal Threshold

Sedative use in OSA has historically been discouraged because of concerns that delayed arousal would produce further blood gas derangement. Furthermore, many of these drugs have myorelaxant properties that could worsen obstruction. However, not all studies show worsening of OSA with sedatives and some show improvement, even in individuals with moderate OSA [99,100]. Moreover, recent studies indicate that certain sedatives might be beneficial for the recruitment of upper airway dilator muscles during sleep [101,102,103]. Therefore, the belief that all sedatives are contraindicated in all patients with OSA is not supported by the literature.

The physiologic basis behind the use of sedatives in OSA can be explained as follows. During an obstructive event, reduced ventilation leads to a buildup in CO_2_ and ventilatory drive that can activate the pharyngeal muscles and reduce upper airway resistance; this process is called pharyngeal muscle compensation. A low respiratory arousal threshold can prevent this mechanism from occurring since patients must remain asleep long enough for these muscles to be recruited [104,105]. Indeed, some studies suggest that airway patency may be restored prior to, or in the absence of, cortical arousal [8,106]. By contrast, a premature arousal may interrupt the sleep continuity and prevent stable breathing from being reached. For this reason, frequent arousals following minor airway collapse is thought to promote cyclic breathing events [107] and ventilatory variability through the ventilatory response to arousal [108,109,110]. They can also reduce the occurrence of slow wave sleep, which is typically a time of stable breathing, even in patients with significant OSA [104,111]. In summary, while arousals help protect people with a high arousal threshold from asphyxia, it may destabilize breathing for patients with a low arousal threshold. Thus, preventing arousals with sedatives in appropriately selected patients with a low arousal threshold could yield more stable breathing and less OSA.

**Figure 4:**
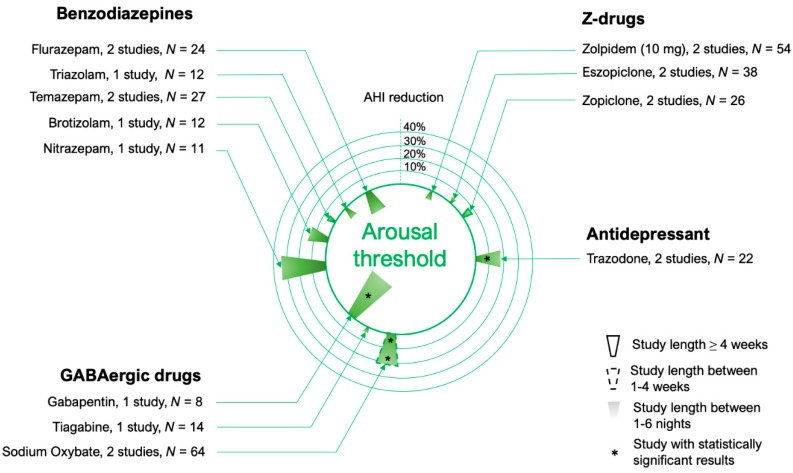
Circular bar plot describing the studies performed using medications that modify the arousal threshold. When multiple doses of a medication were used in different studies, the dose considered is specified in the parenthesis.

It is still not clear if the changes in respiratory arousal threshold can modify the severity of sleep-disordered breathing. While the data collected so far do not suggest that it does (see Figure 4), only a few OSA trials have measured the arousal threshold on and off hypnotics, and more data need to be collected on selected patients with low arousal threshold. Moreover, the drugs tested to date were unable to increase the arousal threshold by more than 20–30% (see below for a detailed description). Ideally, a drug that mimics the effects of slow wave sleep (a state in which the arousal threshold is elevated) would be ideal for reducing OSA severity, given that this is a relatively protected stage of sleep in regards to OSA [111,112,113,114].

### 4.1. Benzodiazepines

Several benzodiazepines have been tested in OSA patients. Cirignotta et al. compared the acute effect of flurazepam 30 mg vs. placebo in two different crossover, placebo-controlled trials [115,116] (total of 24 patients). In one trial, flurazepam significantly worsened the oxygen saturation nadir compared to placebo. Considering both trials, flurazepam caused a non-statistically significant increase in AHI of 16% compared to placebo. In another study, the same investigators tested the effect of brotizolam 0.25 mg vs. placebo [116] and showed no effect on OSA severity.

Barry et al. [117] found that triazolam 0.25 mg significantly increased the arousal threshold by ~20% in 12 patients with severe OSA during a randomized placebo-controlled trial. The AHI in this group remained unchanged, but the obstructive events were longer and led to lower oxygen saturation. Nitrazepam was tested by Hoijer et al. on 11 patients with mild-to-moderate OSA in the doses of 5 and 10 mg; they compared the results to placebo in a one-night crossover study [100]. There was no significant change in AHI or oxygen saturation indices, although there was substantial individual variability in the response: three patients had a worsening of OSA whereas six seemed to improve at the higher dose. Phenotypic traits were not measured so it is uncertain if the improvement was in patients with a low arousal threshold. Overall, there was a sizeable but non-statistically significant reduction in AHI by 33% vs. placebo.

Temazepam was tested in three studies. The first one from Camacho et al. [118] included mild OSA patients ≥60 years of age with insomnia. This was a placebo-controlled randomized trial with two parallel groups and a total of 15 patients. The RDI on temazepam at the dose of 15 or 30 mg was not significantly different from placebo after eight weeks of treatment. The second study by Wang et al. [99] investigated the effect of temazepam 10 mg compared to placebo in a single-night crossover trial including 20 patients, mostly with mild-to-moderate OSA. Once again, the AHI did not change systematically on drug but there was high inter-individual variability in the response. The authors attempted to characterize patients by their baseline chemosensitivity but could not find any relationship between the change in AHI and the baseline response to CO_2_. Arousal threshold was not determined in this study. Lastly, Carberry et al. tested the effect of Temazepam in a four-arm, randomized, placebo-controlled crossover study that assessed several phenotypic variables: upper airway collapsibility, arousal threshold and genioglossus muscle responsiveness. The three hypnotics tested were Zolpidem, Zopiclone and Temazepam. Twenty-one individuals with and without OSA attended an overnight study on placebo and on the drugs. Temazepam did not significantly vary either the arousal threshold or the collapsibility. However, in contrast to conventional wisdom, Temazepam did not adversely affect the genioglossus muscle responsiveness to negative epiglottic pressure swings [119].

### 4.2. Z-Drugs

George et al. tested the effect of zolpidem 10 mg for a single night in a group of 42 patients with an AHI between 10 and 40 events/h in a randomized crossover trial [120]. However, compared to placebo, they found no difference in AHI or oxygen saturation. Zolpidem 10 mg was again recently tested by Carberry et al. [121] in an open label pilot study including 12 OSA patients administered the drug for a single night after a diagnostic polysomnography. There was no systematic effect on oxygen saturation or AHI (which changed minimally from 29 to 33 events/h), but sleep efficiency significantly improved from 77 to 84% on the drug night. In another physiology trial including 21 individuals with and without OSA, the same group recently showed that 10 mg of zolpidem increased the arousal threshold by 25% and, unexpectedly, also the genioglossus response to pharyngeal negative pressure as compared to placebo, suggesting that this medication is at least not harmful for OSA patients [119]. Nonetheless, it is important to notice that a previous trial by Cirignotta et al. [115] testing the effect of zolpidem at the higher dose of 20 mg in a crossover trial of one night in 12 patients showed an increase in the AHI from 17 to 30 events/h (*p* = 0.12), a lowered mean O2 saturation from 91.7 to 88.6% (*p* = 0.02), and a worsened nadir SaO2 from 85.2 to 76.8% (*p* = 0.02).

Among other Z-drugs, eszopiclone and zopiclone were studied in OSA patients to measure their effect on the arousal threshold. Rosenberg et al. [122], in a double blinded crossover trial of two-day treatment, showed that, compared to placebo, eszopiclone did not change the AHI in 21 patients, whereas it improved sleep efficiency and reduced spontaneous arousals. Eckert et al. [123], in a one-night crossover trial including 17 OSA patients, showed that eszopiclone increased the arousal threshold by ~30%. Although the group data did not show a significant reduction in AHI overall, those with a low arousal threshold at baseline (8/17) had their AHI reduced by 43%. Carter et al. [124] showed that zopiclone 7.5 mg administered for one night (*N* = 12) increased the arousal threshold by 20% but did not significantly change the AHI compared to placebo. A subsequent parallel-arm trial from the same group, testing zopiclone (*N* = 14) vs. placebo (*N* = 16), showed a non-significant reduction in AHI between the two groups (−25% from baseline and −15% from placebo) after 30 days of treatment [125]. In the aforementioned four-arm trial by Carberry and coworkers [119] zopiclone 7.5 mg significantly increased arousal threshold (but not AHI) vs. placebo in a group of 21 healthy individuals and OSA patients.

Interestingly, a recent double-blind, placebo-controlled, randomized, cross-over trial from the same group showed that doubling the dose of zopiclone (15 mg) for one night did not have any effect on the arousal threshold (or the AHI), suggesting it is not a dose-dependent effect. On the other hand, this dosage of zopiclone did not affect the next-day sleepiness and alertness measures [126].

### 4.3. Other Hypnotics and Sedatives

Several other sedatives and hypnotics with the potential to increase the arousal threshold have been tested in small trials that, in general, showed minor changes in the arousal threshold and AHI. Tiagabine, a GABA reuptake inhibitor, increased the slow wave activity (sleep depth) but had no effect on the arousal threshold or the AHI [114]. Ramelteon, a melatonin receptors agonist, was tested by Kryger et al. [127] in a group of 26 patients with mild to moderate OSA for one night in a crossover trial and showed no effect on the AHI or oxygen SaO_2_. Melatonin was tested by Deacon et al. [128] in 19 OSA patients over one week of treatment and improved sleep consolidation and mean oxygen saturation but not the AHI.

Gabapentin, an analog of GABA, was recently tested by Piovezan et al. [129] at the dose of 300 mg in a double blind, placebo-controlled, randomized, crossover trial in eight men aged ≥60 years with no or mild sleep apnea. The authors found that the medication significantly increased the mean AHI from 12.4 on placebo to 22.2 (84%) during one-night administration, suggesting a potential deleterious effect of this commonly used drug on the population of older OSA patients.

The acute effect of sodium oxybate, a hypnotic prescribed to patients with narcolepsy, was assessed by George et al. [120] in 42 patients with an AHI between 10 and 40 events/h. In this trial sodium oxybate non-significantly reduced the AHI by 17% from placebo, although the medication significantly increased the number of central apneas and reduced oxygen saturation parameters in three patients, suggesting different individual responses to the treatment. The same authors subsequently performed a two-week parallel-arm trial [130] testing placebo (*N* = 22 OSA patients) vs. sodium oxybate (*N* = 26 OSA patients). This study showed a significant reduction in AHI by 32%. Slow wave sleep was significantly increased by 24%, suggesting that this drug may reduce sleep apnea severity by increasing the time spent in deep sleep and increasing the arousal threshold.

Trazodone is a serotonergic antidepressant with sedative properties. It was tested in several trials on OSA patients as a potential non-myorelaxant hypnotic, and it is currently prescribed as a third-line pharmacologic treatment for chronic insomnia in adults [131]. Heinzer et al. [132] showed that administration of trazodone 100 mg before bedtime to eight OSA patients increased the arousal threshold in response to hypercapnia, allowing tolerance of higher CO_2_ levels without arousal. Eckert et al. [133] showed that trazodone could improve arousal threshold by 30% in seven patients with a low arousal threshold, but it did not affect the AHI compared to placebo. By contrast, Smales et al. [134] showed that trazodone 100 mg reduced the AHI by 26% in 15 unselected patients with an AHI ≥10 events/h, although the arousal threshold was unchanged between nights. Lastly, trazodone 75 mg was used in a pilot study assessing the effects of different behavioral/pharmacological therapies on eight OSA patients. Therapies were chosen based on the baseline pathophysiological causes of OSA and were administered for two weeks: six patients took trazodone alone or in combination with other aids; five of them experienced a decrease in the number of respiratory events/hr (AHI decreased from 26 to 9 in the group as a whole after the therapy). No data related to the arousal threshold on intervention were collected [135].

In summary, hypnotics and sedatives may have different effects on OSA severity but are in general well tolerated and do not increase OSA severity or desaturations if used in normal therapeutic ranges and for a short time. Sodium oxybate and trazodone and some z-drugs may have mild beneficial effects at least in selected OSA patients but more studies need to address this possibility.

## 5. Ventilatory Control (Loop Gain)

Loop gain describes the sensitivity of the negative feedback loop that controls ventilation, which is used to regulate blood gas tension levels within narrow limits [107]. This feedback loop is comprised of different subsystems (i.e., the plant, the controller, and the circulatory delay) that sequentially work to counteract any change in blood gas tension (increase or decrease) due to a corresponding change in ventilation. In general, a high loop gain indicates a control system that disproportionately responds to small changes in PCO_2_, which leads to unstable, periodic breathing (hyperventilation alternating with hypoventilation). When an individual falls asleep, his loop gain physiologically tends to increase slightly [136]. In patients with a collapsible upper airway, large variations in ventilatory drive can increase the propensity for repetitive pharyngeal obstructions in one of two ways. First, the periods of low drive can reduce pharyngeal muscle activation, making the airway floppier and more susceptible to collapse despite low suction pressures. Second, the periods of high drive can produce negative inspiratory pressures that overcome airway dilating forces and suck the airway closed. Either extreme may be detrimental, depending the complex interplay between anatomical and neuromuscular factors within an individual.

**Figure 5:**
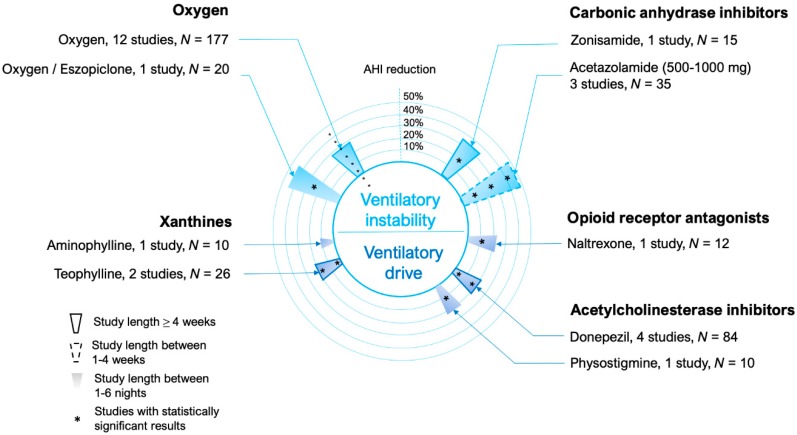
Circular bar plot describing the studies performed using medications that modify ventilatory instability (loop gain) or ventilatory drive. Drugs targeting more specifically ventilatory control instability (loop gain) are represented in the upper part of the circle, those that act more generically as ventilatory stimulants are represented in the lower part of the circle.

The main medical therapies targeting ventilatory instability are listed in Figure 5 and include: (1) carbonic anhydrase inhibitors such as acetazolamide, which has been shown to decrease resting PCO_2_ via generating a transient metabolic acidosis and relative hyperventilation. The lower PCO_2_ will reduce PCO_2_ variations for a given change in ventilation (reduced plant gain = ΔPCO_2_/ventilation) [137]. (2) Oxygen treatment converts the sensed variation of gas tension into a smaller change in ventilatory drive (i.e., decreased chemosensitivity, or controller gain = Δdrive/PCO_2_) [138].

### 5.1. Carbonic Anhydrase Inhibitors

Acetazolamide at the dose of 1000 mg (250 mg four times/day) for two weeks, tested in a randomized placebo-controlled crossover trial in 10 moderate-to-severe OSA patients by Whyte et al. [79], reduced the AHI by 52% compared to placebo. However, it failed to improve symptoms of sleepiness, morning headache and sleep disturbance assessed by a visual analog scale. Tojima et al. [139] tested 250 mg/day of acetazolamide in nine OSA patients for one week and demonstrated a significant AHI reduction of 28% from baseline, which was particularly evident in five patients with more severe symptoms. More recently, Edwards et al. tested the effect of acetazolamide 500 mg twice daily on the OSA phenotypic traits and OSA severity [137]. One-week of therapy with acetazolamide reduced AHI by 52% compared to baseline in the 12 patients enrolled, and it significantly reduced the loop gain by 41%. No other phenotypic trait was modified by the treatment. Our group also demonstrated that acetazolamide may be helpful in OSA by decreasing the ventilatory response to arousals, thus further stabilizing respiration [109].

In a crossover study, Eskandari et al. [140] showed that, in a group of 13 patients with OSA and hypertension, acetazolamide in doses ranging from 500 to 750 mg according to patients’ tolerability reduced the AHI by 40% from baseline over a two-weeks treatment period. This study also involved a second arm in which acetazolamide was administered with CPAP, as well as a third arm with CPAP alone. As expected, the arm in which the combination of acetazolamide and CPAP were given together had the greatest improvement in AHI. However, only in the patients enrolled in the two arms receiving acetazolamide did a significant reduction in mean arterial pressure occur.

Zonisamide is another carbonic anhydrase inhibitor that can also promote weight loss. It was tested vs. placebo and CPAP in a parallel arm trial. Although less efficacious than CPAP, zonisamide at the dose of 100 mg three times daily (*N* = 13) reduced the AHI by ~30% compared to placebo (*N* = 15) during a four-week treatment period. Zonisamide also caused a small but significant weight reduction compared to CPAP after 24 weeks of treatment.

### 5.2. Oxygen

The first studies reporting oxygen administration to OSA patients were in the 1980s and results are inconsistent, likely due to the small sample size and differences in patient selection. Kearley and coworkers [141] initially obtained a lower desaturation index with oxygen administration vs. sham air (split night design) in 11 patients with overlap syndrome (OSA + chronic obstructive pulmonary disease), but no change in AHI was reported. Smith et al. found that, in 12 sleep apnea patients undergoing a randomized protocol, breathing 3 L/min of oxygen vs. air increased oxyhemoglobin saturation and decreased the total AHI, with a relative increase in obstructive events [142]. More recently, Pokorski et al. [143] performed a single-blinded study administering room air or 30% oxygen to 5 OSA patients who did not respond to CPAP and showed a decrease in AHI from 53 to 39 events/h. Landsberg and coworkers [144] explored the effect of 4 L/min of oxygen administered for 30 days to 21 OSA patients who had baseline saturations below 90% and were not suitable to CPAP therapy. In this study, the treatment alleviated OSA-related symptoms but left the RDI unchanged. Multiple randomized trials comparing the effect of oxygen to CPAP and sham air for variable duration (2 weeks to 3 months) were performed [145,146,147,148,149]. Only one of these, a randomized, controlled, three-parallel arm trial [147], showed a favorable effect of 3L/min of oxygen after 2 weeks of treatment compared to baseline in 13 moderate to severe OSA patients, modestly reducing AHI to 44 from 61; no difference was observed compared to the sham air arm (15 patients). These findings highlight the limited usefulness of oxygen at decreasing OSA severity. Additionally, one concern with oxygen in therapy in OSA is that it might lengthen the event duration [150], therefore leading to excess in CO_2_ retention and more marked symptoms.

However, since oxygen has ventilatory stabilizing properties, mainly related to a reduction in peripheral chemosensitivity [151], it would theoretically be a potential treatment option for OSA patients with a higher than normal loop gain. Wellman et al. [138] measured the effect of supplemental oxygen on OSA severity after separating patients based on ventilatory sensitivity. Twelve patients were allocated into two different groups, a high loop gain group (*N* = 6) and a low loop gain group (*N* = 6). In those patients with a high loop gain, administration of oxygen (3 to 5 L/min) caused a reduction in AHI by 53% compared to room air. Conversely, in those with a low loop gain, the AHI was only reduced by 8%. Sands et al. [152] however recently showed that other endotypic traits, in addition to loop gain, may be important in identifying oxygen responders. In a crossover trial including 36 OSA patients, the presence of higher loop gain, milder collapsibility and greater upper airway muscle compensation were the best predictors of successful OSA treatment with oxygen administration (FIO_2_ 40%). In the group of responders (9/36) the AHI dropped by 59% compared to the night when sham air was administered. Edwards et al. [153] also assessed the effect of the combination of 40% oxygen and eszopiclone 3 mg vs. room air + placebo in 20 OSA patients. Such treatment, administered for a single night, reduced the AHI by 43%, and responders to therapy were found to have a less collapsible upper airway, greater pharyngeal muscle compensation, and less severe OSA. These studies point out the importance of oxygen as a potential therapeutic resource for selected OSA patients, conversely, in general OSA population, oxygen administration might be detrimental.

### 5.3. Carbon Dioxide Rebreathing

The use of CO_2_ to stabilize periodic breathing abnormalities is not new, [154] and there have been multiple recent, mostly successful, attempts to do so [155,156]. The extent to which periodic breathing contributes to OSA in some individuals suggests that CO_2_ rebreathing may theoretically have beneficial effects in these patients as well [157]. Xie et al. [158] tested this hypothesis in 26 severe OSA patients and found that prevention of transient hypocapnia could attenuate OSA in selected patients. Indeed, with isocapnic rebreathing (i.e., CO_2_ was added only during hyperpnea to prevent transient hypocapnia) 14 patients identified as having high controller and plant gains reduced their AHI to 31% of control, whereas the 12 with low gains did not show any improvement. This same study showed that hypercapnic rebreathing (i.e., +4.2 mmHg PetCO_2_) reduced the AHI to 15% of control (*N* = 17). Therefore, CO_2_ rebreathing seems to be a promising treatment if patients with high loop gain are identified [136]. Furthermore, it might be useful in NREM isolated OSA, where loop gain seems to have a predisposition to be higher [159]. However, iso/hypercapnic rebreathing administration is still unpractical and difficult to replicate outside of the laboratory.

## 6. Other Drugs Acting on Ventilatory Drive

### 6.1. Xanthines

Xanthines such as aminophylline, theophylline or caffeine increase ventilation by antagonizing adenosine in the central nervous system and improving diaphragm contractility [160]. While xanthines have been used effectively to reduce central apneas in premature infants [161] and patients with periodic breathing and heart failure [162], less consistent results have been obtained in patients with OSA. Mulloy and McNicholas [163] performed a one-month crossover trial on 12 patients with moderate-to-severe OSA. They tested the effect of theophylline 800 mg vs. placebo and found a significant but mild reduction in obstructive events (17% from placebo). Hein et al. [164] tested theophylline for one-week in 14 mild OSA patients and demonstrated an AHI reduction of 27% compared to placebo. Espinoza et al. [165] showed no effect of aminophylline infusions (versus saline) on OSA severity in a group of 10 patients in a one-night crossover trial. Given the inconsistent rate of the findings and the small therapeutic window of the xanthines, these drugs are not recommended for the treatment of OSA.

### 6.2. Opioid Antagonists

Opioid antagonists have shown inconsistent results in several small trials: Guilleminault and Hayes [166] found no effect of naloxone on OSA severity in 28 patients; Atkinson et al. [149,167] improved sleep oxygenation after intravenous injection of naloxone in a group of 10 obese OSA patients; Diamond et al. [168], in a small pilot study on four patients with OSA, found again no effect on AHI. Naltrexone 50 mg administered orally was studied in a crossover trial by Ferber et al. [169] in 12 OSA patients (AHI > 10 events/h). Compared to baseline, the drug modestly reduced the AHI by 12% but improved oxygen indices. Considering the side effects of opioids and the very modest results obtained with some of them at reducing AHI, this treatment is not indicated for OSA.

### 6.3. Acetylcholinesterase Inhibitors

Donepezil is a reversible inhibitor of the acetylcholinesterase enzyme that enhances cholinergic transmission to both muscarinic and nicotinic receptors. Based on the involvement of cholinergic systems in ventilatory control during sleep, and on the evidence that reduced thalamic cholinergic activity was associated with OSA severity in patients with multisystem atrophy, donepezil was initially tested on OSA severity in a group of 11 patients with Alzheimer’s disease and compared with placebo (*N* = 12) in a parallel arm trial by Moraes et al. [170]. Donepezil 10 mg showed a significant reduction in AHI by ~50% in this population over three months of treatment. A subsequent trial by Sukys-Claudino et al. [171], testing the same medication in 11 OSA patients without Alzheimer’s disease for one month, showed an AHI reduction of 23% from baseline and 39% compared to placebo. There were also improvements in oxygen saturation and sleepiness, but sleep efficiency was reduced on the medication. In a parallel group trial performed by Hunchaisri et al. [172], 21 patients taking donepezil for two months were compared to 20 patients taking placebo. There was no significant reduction in AHI on drug compared to placebo and no improvement in oxygen saturation or subjective sleepiness. In a single night, double-blinded, crossover trial on 41 sleep apnea patients, Li et al. [173] tested the effect of donepezil on OSA severity, arousal threshold and loop gain. They found no significant effect on any of these traits as a group. The authors found in a post-hoc analysis that those with a higher loop gain on placebo had a significant reduction in loop gain on donepezil, although this finding may be due to a ‘regression to the mean’ phenomenon. Finally, Hedner et al. [174] tested a continuous infusion of physostigmine, another acetylcholinesterase inhibitor, vs. placebo in a one-night crossover study on 10 subjects with OSA. They found a 24% reduction in AHI on drug, which was more prominent during REM sleep, and a trend for improvement in oxygen levels during sleep.

## 7. Conclusions

In the last 40 years, there have been several attempts to find a pharmacotherapy for OSA with limited success. The research in this field has improved in the last decade thanks to important discoveries in both the pathophysiology of sleep-disordered breathing and basic science. The recent improvements in technologies along with the possibility to apply this knowledge in the clinical setting could allow the adoption of more precise therapies. Although better pharmacologic agents are still needed for effectively raising the arousal threshold, existing drugs that target upper airway muscle activation and loop gain have already brought some encouraging results. Furthermore, the use of combined therapies targeting more traits simultaneously may soon become a successful strategy. In the end, however, larger trials are needed before translating these therapies into the clinical setting.
